# Age and gender specific estimation of visceral adipose tissue amounts from radiological images in morbidly obese patients

**DOI:** 10.1038/srep22261

**Published:** 2016-03-24

**Authors:** Nicolas Linder, Alexander Schaudinn, Nikita Garnov, Matthias Blüher, Arne Dietrich, Tatjana Schütz, Stefanie Lehmann, Ulf Retschlag, Thomas Karlas, Thomas Kahn, Harald Busse

**Affiliations:** 1 Department of Diagnostic and Interventional Radiology, Leipzig University Hospital, Liebigstrasse 20, Leipzig, Germany; 2 Integrated Research and Treatment Center (IFB) Adiposity Diseases, Leipzig University Medical Center, Leipzig, Germany; 3 Department of Internal Medicine, Neurology and Dermatology, Division of Endocrinology and Nephrology, Leipzig University Hospital, Leipzig, Germany; 4 Department of Visceral, Transplantation, Thoracic and Vascular Surgery, Division of Bariatric Surgery, Leipzig University Hospital, Leipzig, Germany; 5 Department of Internal Medicine, Neurology and Dermatology, Division of Gastroenterology and Rheumatology, Leipzig University Hospital, Leipzig, Germany

## Abstract

Image-based quantifications of visceral adipose tissue (VAT) volumes from segmented VAT areas are increasingly considered for risk assessment in obese patients. The goal of this study was to determine the power of partial VAT areas to predict total VAT volume in morbidly obese patients (BMI > 40 kg/m^2^) as a function of gender, age and anatomical landmarks. 130 morbidly obese patients (mean BMI 46.5 kg/m^2^; 94 females) underwent IRB-approved MRI. Total VAT volumes were predicted from segmented VAT areas (of single or five adjacent slices) at common axial landmark levels and compared with the measured ones (V_VAT-T_, about 40 slices between diaphragm and pelvic floor). Standard deviations *σ*_1_ and *σ*_5_ of the respective VAT volume differences served as measures of agreement. Mean V_VAT-T_ was 4.9 L for females and 8.1 L for males. Best predictions were found at intervertebral spaces L3-L4 for females (*σ*_5_ = 688 ml, *σ*_1_ = 832 ml) and L1-L2 for males (*σ*_5_ = 846 ml, *σ*_1_ = 992 ml), irrespective of age. In conclusion, VAT volumes in morbidly obese patients can be reliably predicted by multiplying the segmented VAT area at a gender-specific lumbar reference level with a fixed scaling factor and effective slice thickness.

Obesity is a worldwide increasing healthcare problem. In the United States, for example, over two thirds of the adult population are either overweight (33%, BMI: 25–30 kg/m^2^), obese (35%, 30–40 kg/m^2^) or morbidly obese (6%, > 40 kg/m^2^)^1^. While prevalence of obesity is still rising, in particular the morbid form^2^, more and more is known about its association with an increased overall mortality, often caused by cardiovascular diseases, diabetes or hypertension^3^,^4^.

Visceral adipose tissue (VAT) on the other hand, through its role as an active endocrine tissue, has been considered to be the most dyslipidemic and atherogenic fat depot in the human body^5^,^6^. There is a considerable interest to identify robust markers that allow one to clinically assess risk factors for obesity and monitor related therapeutic interventions. The amount of VAT is one of the currently most promising parameters in that respect.

Quantification of abdominal VAT volumes by cross-sectional imaging, typically by computed tomography (CT) or magnetic resonance imaging (MRI), however, is generally time-consuming^7^,^8^,^9^. Various methods have already been proposed to estimate total VAT volumes from simple measurements on a limited number of slices. Studies using single or five slice VAT areas for VAT volume prediction have mainly focused on patients with BMI values below 40 kg/m^2^ and data for the morbidly obese are lacking^10^,^11^,^12^,^13^,^14^,^15^,^16^.

The goal of this study was therefore to assess the predictive power of simple VAT areas at various anatomical landmarks and to determine the potential impact of cofactors gender and age in a relatively large number of morbidly obese patients. This work also reports corresponding scaling factors for VAT volume prediction from simple VAT areas that might be valuable for further clinical studies.

## Materials and Methods

### Study population

**Figure 1:**
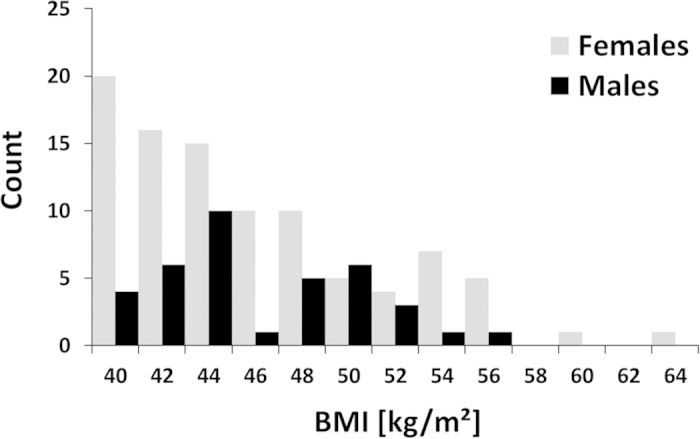
Histogram of BMI distribution for females and males. Mean BMI values were 46.4 ± 5.4 (40.3–64.1) kg/m^2^ and 46.6 ± 4.3 (range 40.1–57.0) kg/m^2^, respectively.

130 morbidly obese patients (18.6–70.7 years old, Caucasian), consisting of 94 females with a mean BMI of 46.4 (range, 40.3–64.1) kg/m^2^ and 36 males with a mean BMI of 46.6 (range, 40.1–57.0) kg/m^2^, were recruited from a dedicated hospital-wide research and treatment program on obesity. Data collection, analysis and publication were approved by the Institutional Review Board (IRB) of the Leipzig University Faculty of Medicine, Leipzig, Germany (reference numbers 283/11-ff, 284/10-ff, 363/10-ff, 363/11-ff) and informed consent was obtained from all subjects. Methods were carried out in accordance with the approved guidelines. Demographic parameters age, weight, height and BMI can be found in Supplementary Table S1. The distribution of BMI values is illustrated in Fig. 1 for both genders.

### Magnetic Resonance Imaging

Patients were examined in a 1.5-T MRI scanner (Achieva XR, Philips Healthcare, Best, Netherlands) in supine position, using the whole-body coil and a two-point Dixon sequence. Data were acquired in two contiguous stacks of 25 images between pelvic floor and diaphragm using a breath-hold technique to reduce motion artefacts. Other imaging parameters were: slice thickness 10 mm, interslice gap 0.5 mm, repetition time TR = 76 ms, echo times TE = 2.3 and 4.6 ms, flip angle = 70°, field of view = 530 mm × 530 mm, acquisition matrix = 216 × 177, reconstruction matrix = 480 × 480, total acquisition time TA = 10 × 13 seconds plus breathing intervals.

### Image Analysis

**Figure 2:**
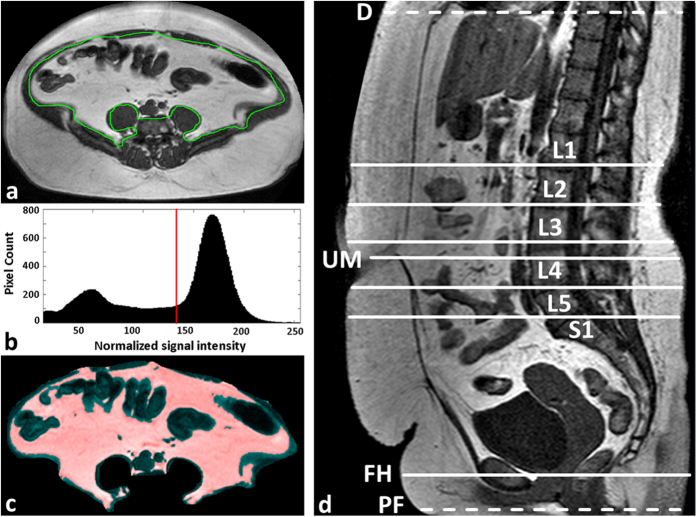
Semi-automated quantification of VAT volume. (**a**) Automatic segmentation and manual correction of the VAT contour (green). (**b**) Histogram of normalized MRI signal intensities (SI) of all pixels within the VAT ROI. All pixels with SI above the predefined but adjustable threshold (red line, typically between lean and fat peaks) are considered as VAT. (**c**) MR image of ROI with VAT pixels overlaid semi-transparently in red. (**d**) Quantitative VAT areas A_VAT_ were assessed on axial MRI slices at the following reference positions (landmarks): umbilicus (UM), femoral heads (FH), and intervertebral discs L1-L2, L2-L3, L3-4, L4-5 and L5-S1. Total VAT volume (V_VAT_) was analysed between pelvic floor (PF) and diaphragm (D).

MRI data were analysed on a standard PC (Dual-Core CPU, 2.60 GHz, 3.25 GB RAM). Total VAT volume (VAT_T_) was quantified between pelvic floor and diaphragm^17^ using a custom-made, previously described software tool under MATLAB (MathWorks, Natick, MA, USA)^9^. In short, VAT boundaries were segmented automatically in the opposed-phase images and edited manually by two experienced radiology residents where corrections were deemed necessary (Fig. 2a). The histogram of the MRI signal intensities (SI) is computed for each slice and typically shows two distinct peak regions resulting from non-fat and fat tissue contributions with a local minimum in between (Fig. 2b). In a previous analysis of our MRI data^13^, best visual agreement between histogram-based (mask) and anatomical fat regions was often observed (by an expert medical reader) for threshold positions some distance away (towards the “fat peak”) from that minimum. The default SI threshold for the separation of fat from not-fat pixels was therefore set half way between local minimum and “fat peak”. This threshold was manually adjusted whenever the colour overlay of the corresponding VAT mask (Fig. 2c) did not match sufficiently well with the anatomical features. Total VAT volume (V_VAT-T_) was calculated from the segmented VAT areas by multiplying the respective pixel count with the pixel area and with the effective slice thickness.

VAT areas of single or five contiguous axial slices (A_VAT-1_ or A_VAT-5_), centered at specific anatomic landmarks, were considered as potential predictors of V_VAT-T_. The landmarks consisted of five intervertebral spaces between L1 and S1 (L1-L2 to L4-L5 and L5-S1), the umbilicus (UM) as well as the femoral heads (FH) (Fig. 2d). For five-slice analysis, four additional slices were selected symmetrically around the anatomical level of the single-slice approach, except for the femoral head level where all slices were placed above the actual anatomic landmark.

Single-slice VAT areas and summed ones (over five adjacent slices) were then plotted against VAT_T_ for each anatomical landmark and a linear fit through the origin was performed defining specific slopes that can then be taken as fixed scaling or conversion factors *f*_1_ or *f*_5_. For a given number of slices (and a specific landmark), total VAT volumes can then be simply estimated from measured VAT areas according to the following equations:


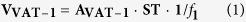



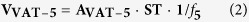


where A_VAT-1_ and A_VAT-5_ are the measured VAT areas (in one and five slices, respectively), ST is the effective slice thickness (nominal slice thickness plus interslice gap), *f*_1_ and *f*_5_ are the above scaling factors and V_VAT-1_ and V_VAT-5_ are the estimated total VAT volumes.

Potential variation of the results with age was addressed by restricting the analysis to three nearly equally sized subgroups for each gender. This resulted in different cut-off ages between male (Group I: < 40.4 years, n = 12, Group II: 41.3–51.3 years, n = 12, and Group III: > 51.3 years, n = 12) and female subgroups (Group I: < 39.2 years, n = 31, Group II: 39.6 – 50.8 years, n = 32, and Group III: > 50.9 years, n = 31).

### Statistical Analysis

Statistical measures of agreement were the coefficient of determination *R*^2^ of a linear fit through the origin and the standard deviations *σ*_1_ or *σ*_5_ of the differences between volume predictions V_VAT-1_ or V_VAT-5_ and actual V_VAT-T_ (Bland-Altman analysis). Correlation between BMI and total VAT volume was analysed with Pearson’s correlation coefficient *r*. All statistical analyses were performed using SPSS 18 (Chicago, IL). Statistical *P* values under 0.05 were considered to be significant.

## Results

Total VAT volumes (V_VAT-T_), single and five-slice VAT areas (A_VAT-1_ or A_VAT-5_) could be determined for all patients and reference points. V_VAT-T_ values in males were significantly higher compared to females (8.1 ± 3.1; range 3.6–15.0 L compared to 4.9 ± 1.7; range 1.4–10.2 L). The correlation between BMI and total VAT volume V_VAT-T_ was poor for both females (Pearson’s *r* = 0.196, *P* = 0.06) and males (Pearson’s *r* = 0.392, *P* < 0.05).

**Figure 3:**
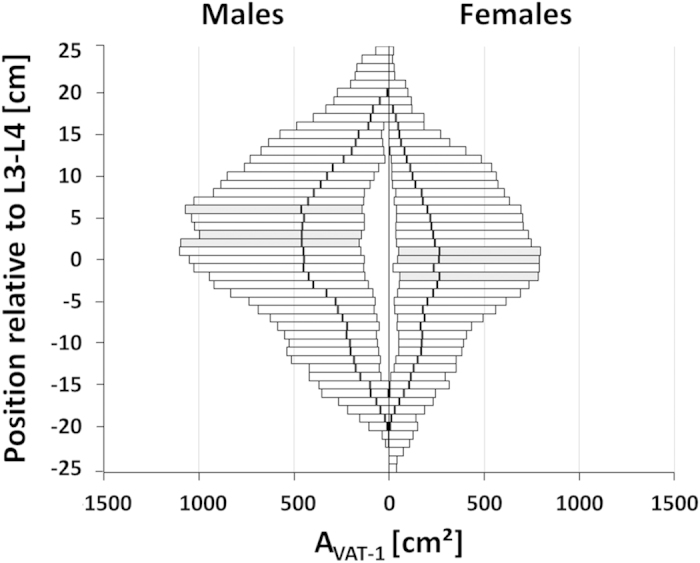
Distribution of visceral fat areas A_VAT-1_ for males (left) and females (right). across all subjects as a function of the axial height *h*_ref_ relative to the reference position at intervertebral space L3-L4 (positive values for more cranial positions). Bars indicate the range between minimum (MIN) and maximum (MAX) A_VAT-1_ values; the bold vertical lines are the median values (MED). Single slice positions with largest *median* VAT areas are highlighted in gray (females: *h*_ref_ = −1.05, 1.05 and 2.1 cm; males: *h*_ref_ = 3.15, 4.20 and 7.35 cm).

A gender-specific distribution of single-slice VAT areas (A_VAT-1_) across the whole study population is illustrated in Fig. 3 for different axial heights *h*_ref_ relative to the reference position at intervertebral space L3-L4 (positive values for more cranial positions). Largest median VAT areas were found at *h*_ref_ = −1.05, 1.05 and 2.10 cm in females and, more cranial, at *h*_ref_ = 3.15, 4.20 and 7.35 cm in males (non-integer values caused by interslice gap of 0.5 mm).

**Figure 4:**
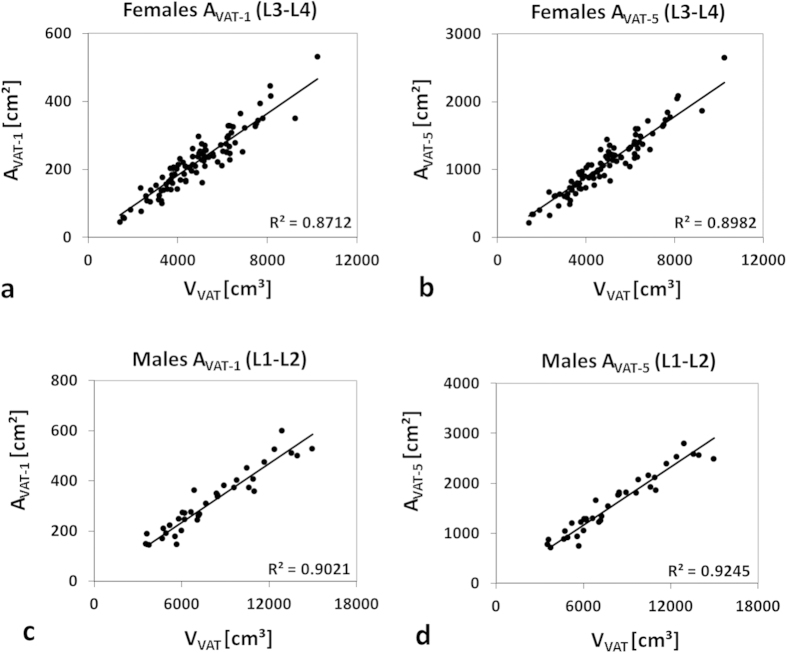
Illustration of (best) agreement between total VAT volume (V_VAT_) and segmented VAT areas (A_VAT-1_ and A_VAT-5_). Solid lines represent linear fits through origin. Best agreement was found in females (**a,b**) and males (**c,d**) at intervertebral levels L3-L4 and L1-L2, respectively. A_VAT-5_ showed slightly better agreement than A_VAT-1_ in both genders.

Coefficients of determination (*R*^2^) between reference V_VAT-T_ and VAT areas (A_VAT-1_ or A_VAT-5_) ranged from 0.48 to 0.90 for males and from 0.44 to 0.89 for females (shown in Supplementary Table S2). Highest values for single-slice estimates were observed at L1-L2 (*R*^2^ = 0.90) in men and at L3-L4 (*R*^2^ = 0.87) in women. Agreement for five-slice estimates was generally better with highest values again at L1-L2 in men (*R*^2^ = 0.93) and at L3-L4 in women (*R*^2^ = 0.89). Sample linear fits through the origin are shown in Fig. 4 for both genders.

Gender-specific scaling factors *f* between A_VAT-1_ or A_VAT-5_ areas and measured V_VAT-T_ at the respective optimum positions were roughly 15% higher for females (one slice: 0.045 vs. 0.039; five slices: 0.222 vs. 0.194). Specific VAT areas (A_VAT-1_ or A_VAT-5_) may then be converted to VAT volume estimates (V_VAT-1_ or V_VAT-5_). Supplementary Fig. S3 shows an example for a substantial VAT difference between two patients with the same BMI.

Bland-Altman analysis revealed mean VAT differences (bias) between −89 and + 260 ml for males and between −82 and + 126 ml for females. As an example, Supplementary Fig. S4 shows two Bland-Altman plots for VAT estimation by five slices around the umbilical and the optimum lumbar level (L3-L4) in females.

**Figure 5:**
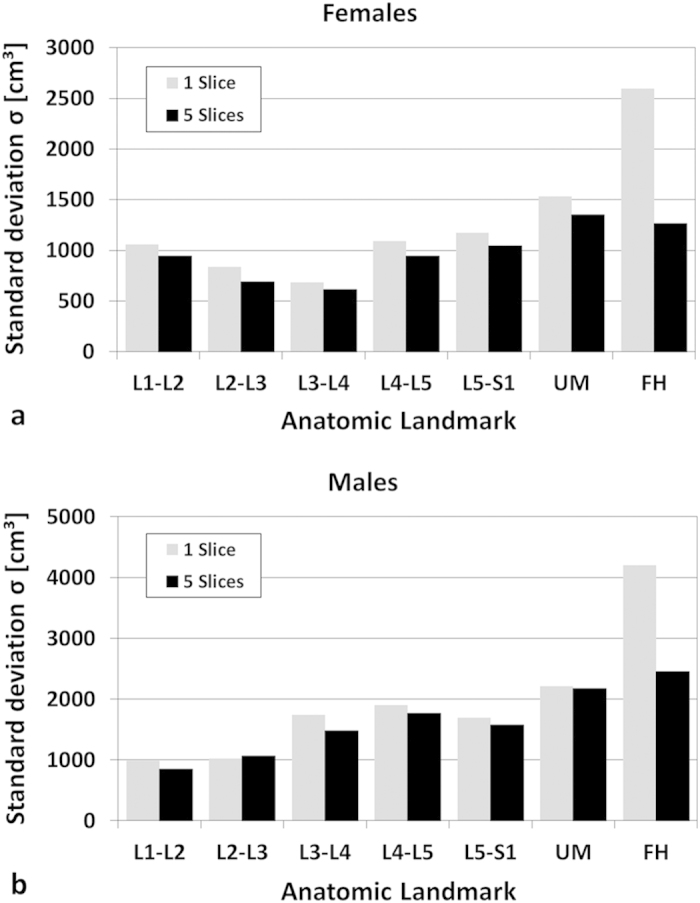
Standard deviations [cm^3^] *σ*_1_ (gray) and *σ*_5_ (black) of the differences between measured (V_VAT-T_) and estimated VAT volumes (V_VAT-1_ and V_VAT-5_). at predefined anatomic reference positions for females (**a**) (n = 94) and males (**b**) (n = 36).

Standard deviations of the differences between measured (V_VAT_) and estimated VAT volumes (V_VAT-1_ and V_VAT-5_) are shown in Fig. 5. Standard deviations *σ*_*1*_ ranged from 685 to 2,595 ml in females and from 989 to 4,204 ml in males while *σ*_*5*_ values ranged from 611 ml to 1,352 ml and from 850 ml to 2,452 ml, respectively. Accuracies varied with anatomical level and showed best agreement at L1-L2 for males with *σ*_*5*_(L1-L2) = 850 ml and *σ*_*1*_(L2-L3) = 989 ml. Minimal standard deviation in females was found at L3-L4 with *σ*_*5*_(L3-L4) = 611 ml and *σ*_1_(L3-L4) = 685 ml. Values of *σ*_*5*_ were generally smaller than *σ*_*1*_, except for level L2-L3 in males. Quantitative agreement at the umbilical level was poor (*σ*_*1*_ = 2,225 and *σ*_*5*_ = 2,186 ml in males; *σ*_*1*_ = 1,533 ml and *σ*_*5*_ = 1,352 ml in females). Largest standard deviations were found at the femoral heads for both settings.

Analysis of three equally sized subgroups showed no major variation of either *σ*, *R*^*2*^ or *f* values with age (shown in Supplementary Table S5). The lowest *σ* values of all age groups were again observed at L3-L4 in females. For single slice analysis in males, subgroups I and II (<51.3 years) showed the smallest *σ* value at L2-L3, slightly lower than that at L1-L2.

## Discussion

We have described and validated a clear-cut method to estimate VAT volumes from segmented VAT areas of a limited number of axial images in morbidly obese patients (mean BMI: 46.6 kg/m^2^). Similar to previous results in less obese patients^10^,^11^,^12^,^13^,^14^,^15^,^16^, best agreement was generally observed for intervertebral spaces above L4-L5 and also depended on gender (here L3-L4 in females and L1-L2 in males). Maislin *et al*.^10^ and Demerath *et al*.^11^, in contrast, did not find any gender effect in their cohorts.

Besides the differences seen between genders, the optimum reference also seems to depend on the degree of obesity (BMI range). While previous data in male patients with BMIs between 25 and 40 kg/m^2^ showed best correlations at L2-L3^13^, the present study suggests a more cranial level (L1-L2) for morbidly obese patients. In females, VAT areas at L3-L4 best predicted total VAT volumes independent of BMI. Both gender and BMI group should therefore be considered for reliable estimation of total VAT volumes.

The slight axial offset between both genders is most likely attributable to a difference in average body shape showing the bulk of the visceral fat at more cranial positions in males (Fig. 3). The higher accuracy could then be simply explained by the larger VAT fraction that is used for computation. In cases, where a gender-specific analysis in morbidly obese patients is not feasible, a single (common) reference might be needed which would then be L2-L3. While this choice will only be second best, the corresponding penalties in accuracy appear to be acceptable.

Anatomic landmarks like the umbilicus or femoral heads provided only poor estimates of VAT volumes. Standard deviations, in particular at the femoral head level were almost twice as large. For the umbilical level, this could be caused by the large positional variation between subjects with respect to skeletal landmarks. For the femoral head level, findings are most likely explained by the relative small VAT amounts in the pelvic cavity that are less representative for the entire VAT volume. Our results agree well with previous ones in overweight to severely obese patients^13^ but disagree with findings by Schwenzer *et al*.^14^ who found relatively strong correlations at the umbilical level. This incoherence can be attributed to the underlying BMI difference (46.6 ± 5.1 here vs. 29.7 ± 5.2 kg/m^2^ in their group) and a different patient position during examination (prone vs. supine).

Analyses within three age subgroups essentially confirmed the gender-specific references found overall. Therefore, we do not see a need for age-specific analyses which is also in line with findings in less obese patients^13^.

The extension to five slices yielded slightly better accuracies at the same best and second-best landmarks which can be simply explained by the larger amount of information contained. A previous five-slice approach in obese adolescents by Springer *et al*.^15^, in contrast, worked reliably for females only and also showed no improvement over single slices. Possible explanations for this incoherence could be a different choice of reference levels (umbilicus and femoral heads vs. intervertebral spaces) or the relatively small number of subjects (n = 40; 22 males).

Correlation of BMI with total VAT volume (both from CT or MRI) is generally poor^14^,^18^,^19^,^20^ mainly because VAT constitutes a much smaller fraction to total adipose tissue than SAT, for example, an average of only 4% (females) and 12% (males) in a recent study of 142 patients^21^. The relatively low correlation coefficients (Pearson’s *r*) computed for our patients (0.32 for females and 0.19 for males) clearly underline the need for dedicated VAT measurements^22^.

Our study focussed on morbidly obese patients with a mean BMI of roughly 47 kg/m^2^ (range 40–64 kg/m^2^). Maislin *et al*.^10^ have partially included such patients for analysis with a maximum BMI of 51.2 kg/m^2^ but a median BMI of 32.5 kg/m^2^ only. Our work provides gender-specific scaling factors *f* for two different slice numbers (1 and 5) and seven common landmarks that can be interpreted as the fraction of the partial slice volumes to the whole abdominal VAT. These factors do not depend on the specific slice thickness and can therefore be easily used by other researchers and clinicians.

One limitation is the retrospective, single-centre design of our study which was effectively determined by the IRB approval of the underlying clinical studies. Our higher number of females simply reflects the percentage upon recruitment and findings in males should therefore be interpreted slightly more carefully than those in females. Although the BMI range studied here makes up a smaller part of the overall population only^1^, those patients are more likely to benefit from a reliable assessment of the risk factors and subsequent treatment. Slice-based VAT assessment is becoming an important diagnostic tool for risk stratification^23^,^24^ but has also been considered for VAT monitoring under various interventions^25^,^26^,^27^,^28^, albeit with less success.

In conclusion, our data in morbidly obese patients suggest that VAT areas should generally be measured at axial heights above L4-L5 (in line with findings in less obese patients), preferentially centered around L3-L4 in females and L1-L2 in males. Umbilical or femoral head levels should not be considered (in neither BMI group). The second-best common reference for both genders was L2-L3. Higher BMI values in males seem to shift that level cranially (from L2-L3 to L1-L2) while that in females remained at L3-L4. We could not identify substantial differences with age but found that the analysis of more than one slice (here 5) will yield slightly better VAT measures. We believe that these findings have a high practical value and may also encourage more clinical work on the role of VAT in obesity, not only for morbid forms of the disease.

## Additional Information

**How to cite this article**: Linder, N. *et al*. Age and gender specific estimation of visceral adipose tissue amounts from radiological images in morbidly obese patients. *Sci. Rep*. **6**, 22261; doi: 10.1038/srep22261 (2016).

## Supplementary Material

Supplementary Information
