# Supplementary material for: Age and gender specific estimation of visceral adipose tissue amounts from radiological images in morbidly obese patients
Source: Sci Rep. 2016 Mar 24;6:22261. doi: 10.1038/srep22261 (PMC4806365; doi:10.1038/srep22261)
Supplement: Supplementary Information [file srep22261-s1.pdf]

Supplementary information

Age and gender specific estimation of visceral adipose tissue amounts from radiological images in morbidly obese patients

Nicolas Linder, MD [1,2,†], Alexander Schaudinn, MD [1,2,†], Nikita Garnov, PhD [1,2], Matthias Blüher, MD [2,3], Arne Dietrich, MD [2,4], Tatjana Schütz, PhD [2,4], Stefanie Lehmann, MPH [2], Ulf Retschlag [2], Thomas Karlas, MD [2,5], Thomas Kahn, MD [1], Harald Busse, PhD[1*]

[1] Department of Diagnostic and Interventional Radiology, Leipzig University Hospital, Liebigstrasse 20, Leipzig, Germany

[2] Integrated Research and Treatment Center (IFB) Adiposity Diseases, Leipzig University Medical Center, Leipzig, Germany

[3] Department of Internal Medicine, Neurology and Dermatology, Division of Endocrinology and Nephrology, Leipzig University Hospital, Leipzig, Germany

[4] Department of Visceral, Transplantation, Thoracic and Vascular Surgery, Division of Bariatric Surgery, Leipzig University Hospital, Leipzig, Germany

[5] Department of Internal Medicine, Neurology and Dermatology, Division of Gastroenterology and Rheumatology, Leipzig University Hospital, Leipzig, Germany

[†] Both authors have equally contributed to this work.

[*] **Correspondence to:** Harald Busse
Department of Diagnostic and Interventional Radiology
Leipzig University Hospital
Liebigstrasse 20
04103 Leipzig, Germany
Phone / Fax: +49 (341) 97-17413 / -17409
Email: harald.busse@medizin.uni-leipzig.de

	Females		Males	
Number of Patients	*n*=94		*n*=36	
Age [years]	44.4 ± 11.4	(18.6 – 70.7)	44.3 ± 10.8	(22.2 – 63.6)
Height [cm]	166.2 ± 7.1	(151 – 186)	179.9 ± 7.8	(162 – 203)
Weight [kg]	128.2 ± 17.4	(94.0 – 168.0)	151.0 ± 17.6	(120.6 – 185.5)
BMI [kg/m²]	46.4 ± 5.4	(40.3 – 64.1)	46.6 ± 4.3	(40.1 – 57.0)

Table S1: Anthropometric data of our study population (n=130) for both genders reported as mean ± standard deviation (range).

	Females		Males	
	R^2	f	R^2	f
A_{VAT-1} (L1-L2)	0.77	0.036	**0.90**	**0.039**
A_{VAT-1} (L2-L3)	0.84	0.042	0.88	0.041
A_{VAT-1} (L3-L4)	**0.87**	**0.045**	0.70	0.040
A_{VAT-1} (L4-L5)	0.72	0.041	0.68	0.032
A_{VAT-1} (L5-S1)	0.67	0.030	0.77	0.023
A_{VAT-1} (UM)	0.44	0.037	0.55	0.030
A_{VAT-1} (FH)	0.85	0.012	0.48	0.008
A_{VAT-5} (L1-L2)	0.80	0.179	**0.93**	**0.194**
A_{VAT-5} (L2-L3)	0.88	0.210	0.86	0.202
A_{VAT-5} (L3-L4)	**0.89**	**0.222**	0.76	0.198
A_{VAT-5} (L4-L5)	0.78	0.203	0.71	0.162
A_{VAT-5} (L5-S1)	0,70	0.156	0.77	0.122
A_{VAT-5} (UM)	0.50	0.186	0.53	0.151
A_{VAT-5} (FH)	0.48	0.092	0.52	0.065

Table S2: Coefficients of determination R^2 of linear regression between segmented VAT areas of single (A_{VAT-1}) or five adjacent slices (A_{VAT-5}) and total VAT volumes (V_{VAT-T}) for different anatomical landmarks (see text for abbreviations) with resulting scaling factors *f*. Corresponding conversion or scaling factors *f* are given for both genders. Bold values indicate best correlation.

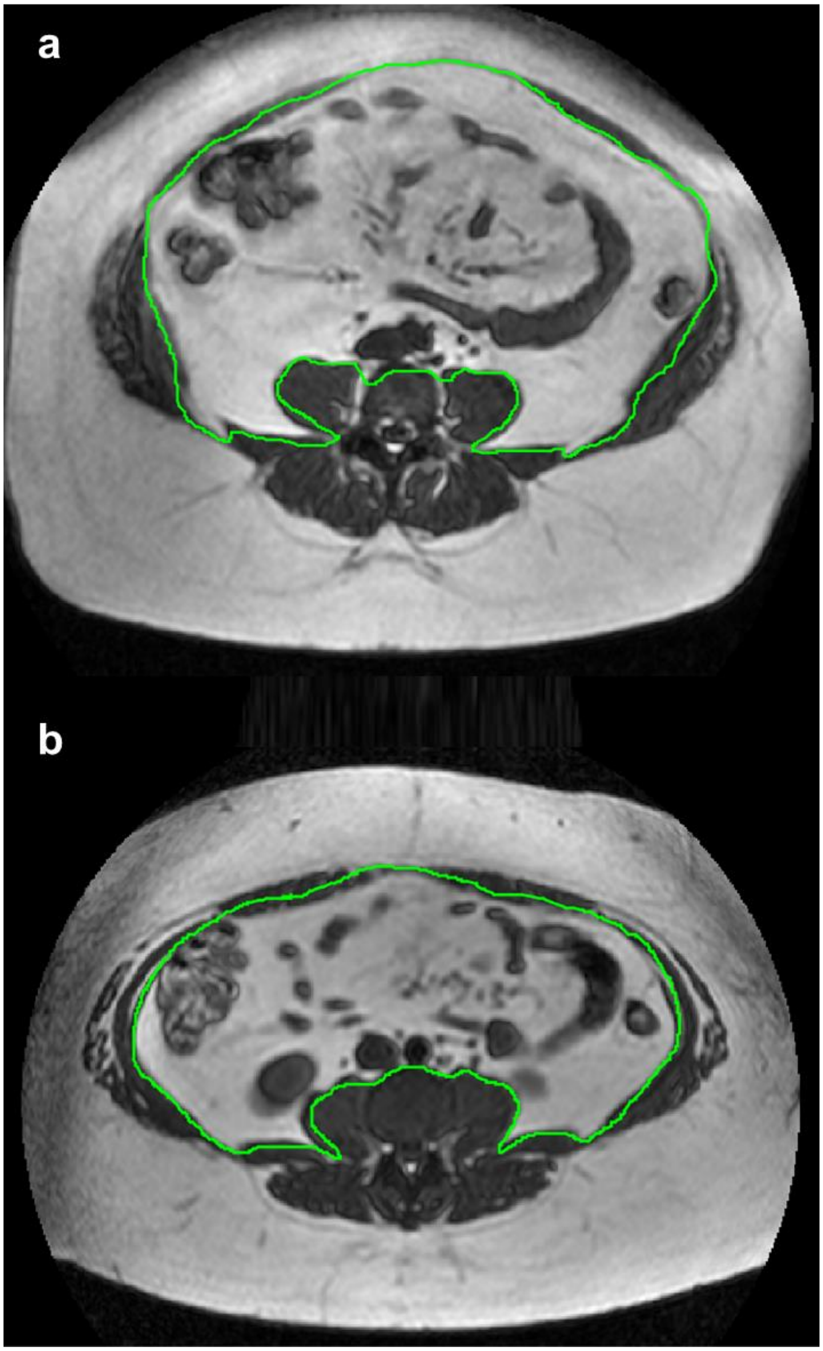

Figure S3: Figure S3: Comparison of MRI-derived VAT amounts at lumbar disc level L3-L4 for two morbidly obese patients "a" and "b" (same gender). Contours denote segmented VAT boundaries. Patient "a" had a 65% larger VAT area A_{VAT-1} and a 55% larger total VAT volume V_{VAT-T} (between diaphragm and pelvic floor) although both patients happened to have the same BMI. Note that SAT is cut off at the edges of the FOV (53 cm).

a

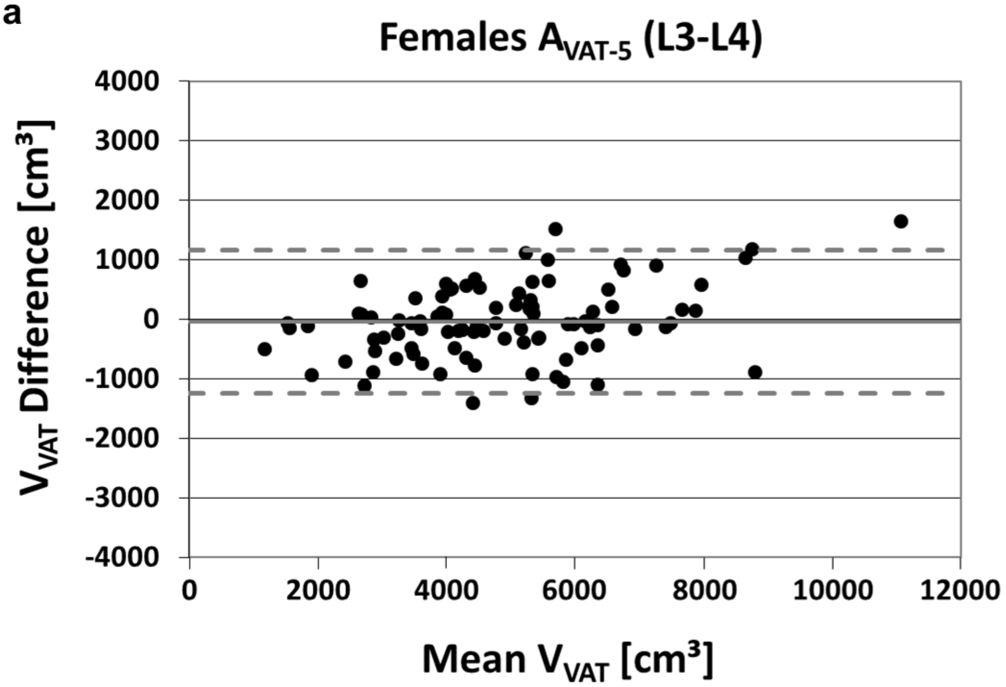

b

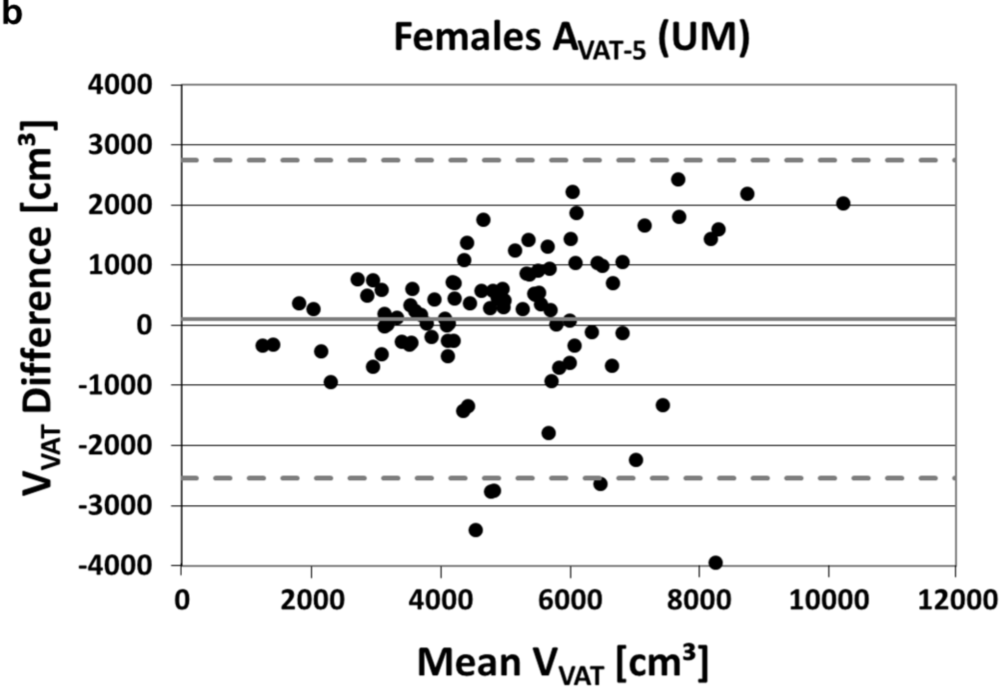

Figure S4: Sample Bland-Altman plots in females (n=94) showing levels of agreement between estimated (V_{VAT-5}) and measured total VAT volumes (V_{VAT-T}) at the optimum L3-L4 (**a**) and the umbilical level (**b**). Solid and dashed gray lines indicate bias and limits of agreement, respectively (here: bias ± 1.96 · σ_5).

Females

Age	All ages		< 39.2 years		39.6 – 50.8 years		> 50.9 years	
Number of patients	*n*=94		*n*=31		*n*=32		*n*=31	
	σ	f	σ	f	σ	f	σ	f
A_{VAT-1} (L2-L3)	836	0.042	802	0.040	907	0.043	791	0.042
A_{VAT-1} (L3-L4)	**685**	0.045	**613**	0.044	**768**	0.045	**654**	0.047
A_{VAT-5} (L2-L3)	691	0.210	629	0.199	747	0.218	655	0.208
A_{VAT-5} (L3-L4)	**611**	0.222	**573**	0.214	**638**	0.221	**603**	0.229

Males

Age	All ages		< 40.4 y.o.		41.3 – 51.3 y.o.		> 51.3 y.o.	
Number of patients	*n*=36		*n*=12		*n*=12		*n*=12	
	σ	f	σ	f	σ	f	σ	f
A_{VAT-1} (L1-L2)	**989**	0.039	854	0.038	845	0.041	**1091**	0.038
A_{VAT-1} (L2-L3)	1023	0.041	**835**	0.039	**825**	0.043	1240	0.040
A_{VAT-5} (L1-L2)	**850**	0.194	**762**	0.189	**651**	0.203	**975**	0.188
A_{VAT-5} (L2-L3)	1063	0.203	802	0.193	922	0.213	1313	0.198

Table S5: Age-dependent analysis for best and second-best anatomical references in both genders for the prediction of V_{VAT-T} from segmented VAT areas A_{VAT-1} and A_{VAT-5} as well as corresponding scaling factors f. Bold values correspond to best agreement (smallest standard deviation σ) of all landmarks within each age group. L1-L2 to L3-L4: lumbar intervertebral spaces.
